# Occupational transmission and prevention of HBV among health care workers in a tertiary care center

**DOI:** 10.1186/1471-2334-12-S1-P33

**Published:** 2012-05-04

**Authors:** Ravikumar Mantri, Febe Ranjitha Suman, R Krishnamoorthy, Vinodkumar Panicker

**Affiliations:** 1 Department of Transfusion Medicine, Sri Ramachandra University, Porur, Chennai, India

## Background

Every day health care workers are exposed to dangerous and deadly blood borne pathogen like HBV. HBV is 100 times more likely to be transmitted after a percutaneous exposure through infected blood than HIV. Hepatitis B infection can be prevented by vaccination.

## Aim of the study

To study the effect of vaccination program among the health care workers of Sri Ramachandra University (SRU), Porur, Chennai. It is mandatory for all health care workers of SRU to be vaccinated against HBV at the time of appointment, producing anti-HBs titer is mandatory. They are advised for re-vaccination according to their anti-HBs titer.

## Methodology

This study was conducted among the health care workers of SRU who had needle stick injury for a period of 1 year (1st November 2010 to 31st October 2011).The blood samples were processed for serology screening by Micro Particle Enzyme Immuno assay (MEIA) followed by ELISA. The results were studied.

## Result

85 samples from health care workers who had needle stick injury were processed .The samples were from doctors (38%), nurses (20%), technicians (7%), housekeeping staff (23 %), and students (10%). All the samples of injured persons were found seronegative by both methods, anti-HBs titer was found to be protective.

## Conclusion

Strict HBV vaccination program and monitoring the anti-HBs titer were effective in prevention of HBV transmission by needle stick injury to health care workers of SRU. This can be implemented in all health care organizations for the benefit of health care providers.

